# Undiagnosed Pulmonary Sequestration in a Young Adult Presenting As Necrotizing Pneumonia and Sepsis

**DOI:** 10.7759/cureus.109172

**Published:** 2026-05-19

**Authors:** Julia Eazer, Nicholas Chin, Jacob Kalin, Karim Hanna

**Affiliations:** 1 Medicine, University of South Florida Morsani College of Medicine, Tampa, USA; 2 Dermatology, University of South Florida Morsani College of Medicine, Tampa, USA; 3 Family Medicine, University of South Florida, Tampa, USA

**Keywords:** congenital lung malformation, intralobar pulmonary sequestration, necrotizing pneumonia, pulmonary infection, sepsis

## Abstract

Pulmonary sequestration is a rare congenital lung abnormality in which a portion of nonfunctioning lung tissue does not communicate with the tracheobronchial tree. Additionally, it receives its blood supply from systemic circulation rather than the pulmonary arteries. Although it is often diagnosed in childhood, some patients remain asymptomatic for years and are not identified until later in adolescence or adulthood. In this case, a 21-year-old male patient with no prior medical history presented with sepsis due to necrotizing pneumonia. His symptoms developed over several weeks and included recurrent respiratory complaints, weight loss, and acute chest and abdominal pain. CT angiography demonstrated an 8 × 9 cm microcystic lesion in the left lower lobe. The lesion was supplied by the descending thoracic aorta and drained into the pulmonary artery, findings consistent with intralobar pulmonary sequestration. Surrounding cavitary and ground-glass changes suggested a superimposed necrotizing infection. He was treated with broad-spectrum antibiotics along with supportive care and nutritional supplementation for associated protein-calorie malnutrition. Because of ongoing inflammation and concern for operative risk, thoracic surgery recommended delaying resection, with a plan for an interval left lower lobectomy once the infection had resolved. This case reflects a less typical presentation of pulmonary sequestration complicated by severe infection. It also serves as a reminder to consider congenital lung anomalies in young adults with recurrent or unexplained pulmonary infections, and highlights the importance of appropriate imaging and multidisciplinary coordination when determining management.

## Introduction

Pulmonary sequestration is an uncommon congenital abnormality arising from aberrant foregut budding during embryologic development, resulting in a segment of nonfunctional lung tissue that lacks communication with the tracheobronchial tree and receives its arterial blood supply from the systemic circulation, most often the thoracic or abdominal aorta [1,2]. It is broadly classified into intralobar and extralobar types. Intralobar sequestration, the more common variant, shares a pleural covering with adjacent lung parenchyma and typically presents later in adolescence or adulthood. In contrast, extralobar sequestration has a separate pleural investment, is more often identified in infancy, and is less frequently associated with infection [2].

Many patients remain asymptomatic for years, particularly in cases of intralobar sequestration [2,3]. When detected later in life, the condition is frequently identified incidentally on chest imaging, although it may also present with recurrent pulmonary infections due to impaired drainage and abnormal vascular supply of the sequestered lung tissue [3,4]. The presence of systemic arterial supply is clinically significant, as it contributes to both increased susceptibility to infection and increased risk of intraoperative hemorrhage, complicating surgical management [4].

While recurrent infections, lung abscess, and empyema have been described in pulmonary sequestration, reports of necrotizing pneumonia as the initial presentation are limited, and progression to sepsis in a previously healthy young adult has not been well characterized in the literature [4,5]. This distinction is important because it indicates a more severe and atypical disease course that may delay diagnosis and complicate management. Here, we describe a previously healthy male patient who developed sepsis from necrotizing pneumonia arising within sequestered lung tissue. This case emphasizes the importance of early imaging in atypical pulmonary infections and highlights the diagnostic and therapeutic challenges associated with severe infection in the setting of congenital pulmonary anomalies.

## Case presentation

**Figure 1:**
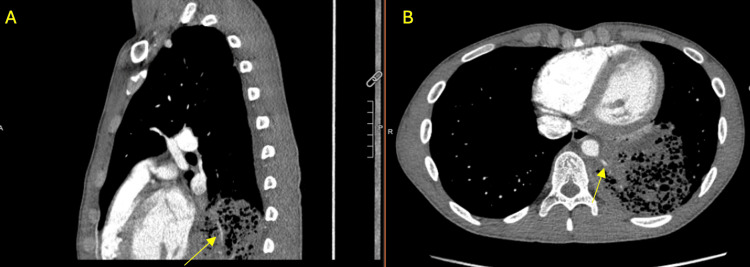
Intralobar pulmonary sequestration with cavitary lesion and systemic arterial supply (A) Sagittal view demonstrating the cavitary lesion and its vascular supply. (B) Axial (transverse) view highlighting the relationship between the lesion and the surrounding lung parenchyma. Yellow arrows indicate aberrant arterial branches arising from the thoracic aorta supplying the sequestration

A 21-year-old male patient with no significant past medical history presented to an outside hospital emergency department with a two-day history of left-sided chest and abdominal pain that began after swimming. He also reported shortness of breath, productive cough, subjective fever, and an episode of lightheadedness concerning for near-syncope during this period. Over the preceding three weeks, he experienced multiple upper respiratory illnesses that he self-treated. Additionally, he reported an unintentional 30-pound weight loss over the past several months. Vital signs were notable for tachycardia (heart rate 110 bpm), hypotension (BP 107/63 mmHg), and oxygen saturation of 100%. Physical examination demonstrated decreased breath sounds at the left lung base, temporal wasting, and reduced subcutaneous fat with a BMI below 19, consistent with protein-calorie malnutrition. Labs at the outside hospital demonstrated a markedly elevated WBC of 37.33 × 10³/µL with a neutrophil predominance of 82% (absolute neutrophil count 30.61 × 10³/µL), bandemia at 6% (absolute band count 2.24 × 10³/µL), and lymphopenia at 5%. Toxic granulation was present on the peripheral smear. Hemoglobin was 12.3 g/dL and a normal mean corpuscular volume of 88.9 fL, consistent with normocytic anemia of acute inflammation. Lactate was mildly elevated at 2.3 mmol/L. D-dimer was 1.3. Total bilirubin was 1.5, and total protein was 8.4, with the remainder of the comprehensive metabolic panel within normal limits. CRP, ESR, and procalcitonin were not obtained. An arterial blood gas obtained on the afternoon of admission demonstrated a pH of 7.39, a partial pressure of carbon dioxide of 41 mmHg, a partial pressure of oxygen of 29 mmHg, an O_2_ saturation of 41%, a bicarbonate of 25 mmol/L, a lactic acid of 1.8 mmol/L, and a hemoglobin of 11.8 g/dL. CT imaging revealed a sharply demarcated 8 × 9 cm microcystic lesion in the left lower lobe with feeding arteries arising from the descending thoracic aorta and venous drainage into the pulmonary artery (Figures 1A, 1B), with surrounding ground-glass opacities and cavitary changes concerning for superimposed infection.

Given the patient’s significant weight loss and subacute symptoms, the differential diagnosis included tuberculosis, malignancy, and chronic pulmonary infection. He met systemic inflammatory response syndrome criteria and was diagnosed with sepsis secondary to necrotizing pneumonia involving sequestered lung tissue [6]. Infectious workup was initiated on admission and included blood cultures, sputum cultures, QuantiFERON-TB Gold (QIAGEN, Hilden, Germany), HIV, and a respiratory viral panel. HIV and the respiratory viral panel returned negative. The QuantiFERON-TB Gold result was indeterminate. Blood and sputum cultures demonstrated no growth. Empiric antibiotic therapy with cefepime 1 g IV was given once. Following this workup, he was transferred to a tertiary academic center for further management.

On admission to the hospital, ceftriaxone 1 g IV and vancomycin 1,500 mg IV were administered, given concern for resistant organisms in the setting of sepsis. Acetaminophen, ibuprofen, and PRN oxycodone were initiated for symptom control. Nutritional support, including oral supplements and daily calorie tracking, was also initiated, given evidence of malnutrition. On the second day of admission, a repeat CBC showed a significant decline in white blood cell (WBC) count to 16.08 × 10³/µL from the admission value of 37.33 × 10³/µL, indicating a rapid early response to antibiotic therapy. However, bandemia worsened to 24% (absolute band count 3.86 × 10³/µL) with the appearance of metamyelocytes at 4%, and lymphopenia persisted at 4%, reflecting ongoing left shift and systemic inflammation. Hemoglobin declined to 9.8 g/dL. Detailed laboratory trends and antimicrobial management throughout hospitalization are summarized in Table 1. Additional infectious workup obtained that day included urine Legionella antigen, urine *Streptococcus pneumoniae* antigen, and methicillin-resistant *Staphylococcus aureus* nares swab, all of which returned negative. Lower respiratory sputum Gram stain revealed many WBCs with polymorphonuclear predominance, few epithelial cells, and mixed Gram-positive and Gram-negative organisms consistent with normal respiratory microbiota, without isolation of a dominant pathogen. Given the negative microbiological data and the clinical picture, antibiotic therapy was narrowed. Piperacillin-tazobactam (Zosyn) 3.375 g IV q6h was started and continued alongside vancomycin 1,000 mg q8h. Thoracic surgery was consulted and confirmed the diagnosis of intralobar pulmonary sequestration complicated by necrotizing pneumonia. Given active inflammation and concern for intraoperative bleeding from aberrant systemic vessels, surgical resection was deferred until the acute infection had improved.

**Table 1:** Integrated timeline of laboratory parameters and antimicrobial therapy in necrotizing pneumonia complicating pulmonary sequestration Laboratory trends (white blood cell count, absolute neutrophil count, bandemia, and hemoglobin) are presented in conjunction with clinical status and antimicrobial management. Initial severe leukocytosis with neutrophilic predominance improved following initiation of empiric antibiotics, though persistent inflammatory markers suggested ongoing necrotizing infection. Antibiotic adjustments were made in response to clinical progression and negative microbiological results, with eventual transition to oral therapy prior to discharge. Arrows (↑, ↓) indicate directional change relative to the prior measurement WBC: white blood cell count; OSH: outside hospital; ANC: absolute neutrophil count; Hgb: hemoglobin

Hospital day	Key lab values	Clinical interpretation	Antibiotics and intervention
Day 1 (admission)	WBC 37.33	Severe leukocytosis with early sepsis	Cefepime 1 g IV (OSH) → Ceftriaxone 1 g IV + Vancomycin 1,500 mg IV (administered after initial labs)
	ANC 30.61		
	Bands 6%		
	Hgb 12.3		
	Lactate 2.3		
Day 2	WBC 16.08 ↓	Rapid WBC improvement, worsening left shift → ongoing inflammation	Transition to piperacillin-tazobactam 3.375 g IV q6h + vancomycin
	Bands 24% ↑		
	Metamyelocytes 4%		
	Hgb 9.8 ↓		
Day 3	WBC 12.10 ↓	Continued response, persistent neutrophilia	Continue Zosyn + vancomycin
	Neutrophils 82.9% ↑		
	Hgb 9.5 ↓		
Day 4	WBC 13.26 ↑	Persistent inflammatory response	Vancomycin dose adjusted (1,250 mg), continue Zosyn
	Bands 16% ↓		
	Hgb 9.2 ↓ (nadir)		
Day 5	WBC 14.72 ↑	Stabilizing leukocytosis	Continue Zosyn + vancomycin
	ANC 10.89 ↓		
	Hgb 9.6 ↑		
Day 6	WBC 15.70 ↑	Persistent but improving inflammation	Zosyn discontinued; vancomycin completed → start doxycycline 100 mg PO q12h
	ANC 13.03 ↑		
	Hgb 10.4 ↑		
Day 7 (discharge)	WBC 15.21 ↓	Clinically stable, residual inflammation	Discharged on amoxicillin-clavulanate 875/125 mg PO q12h + doxycycline 100 mg PO q12h (15 days)
	Hgb 10.2 ↓		

By hospital day 3, leukocytosis continued to improve following initiation of antibiotics, though neutrophilic predominance and lymphopenia persisted. Hemoglobin declined further, consistent with ongoing inflammation. On hospital day 4, there was a slight increase in the WBC count, with persistent bandemia, indicating a continued inflammatory response despite overall clinical improvement. Over the remainder of hospitalization, leukocytosis persisted but stabilized in the mid-teen range. Neutrophilia and lymphopenia remained present, while hemoglobin levels stabilized after reaching a nadir earlier in the admission. Overall, the marked reduction in WBC from admission was consistent with a response to antibiotic therapy, with residual leukocytosis attributed to ongoing inflammation from the necrotizing process rather than treatment failure. On day 6, piperacillin-tazobactam and vancomycin were discontinued. Oral doxycycline 100 mg q12h was initiated in preparation for transition to outpatient therapy. The patient was discharged on hospital day 7 in stable condition. Discharge antibiotics included a 15-day course of both amoxicillin-clavulanate (augmentin) 875-125 mg PO q12h and doxycycline 100 mg PO q12h. He was scheduled for outpatient follow-up with thoracic surgery for a planned interval left lower lobectomy once the infection had fully resolved.

The patient underwent thoracotomy with left lower lobectomy 17 days after discharge without complications. Pathologic evaluation of the resected specimen was negative for acid-fast bacilli on AFB staining and negative for fungal organisms on Grocott’s methenamine silver histochemical staining. Multiple benign reactive regional lymph nodes were identified. Intraoperative cultures were sent per infectious disease recommendations and returned negative, with the causative organism presumed to have been eradicated by the completed antibiotic course. The patient remained clinically stable on subsequent outpatient follow-up.

## Discussion

Pulmonary sequestration accounts for fewer than 6% of congenital pulmonary malformations and is classified as intralobar or extralobar based on pleural investment [7]. Intralobar sequestration, as seen in this patient, is the more common subtype and typically presents later in life. More than half of cases are discovered incidentally on imaging; when symptoms occur, recurrent pulmonary infections are the most frequent presentation due to impaired drainage of sequestered lung tissue [8]. The abnormal systemic arterial supply and lack of communication with the tracheobronchial tree contribute to ineffective clearance of secretions and impaired host defense, predisposing the sequestered segment to recurrent infection [8]. In more severe cases, this can progress to abscess formation, necrosis, or, rarely, necrotizing pneumonia, as demonstrated in this case. The extent of necrosis observed here likely reflects a prolonged, subacute infectious process, consistent with the patient’s history of progressive symptoms and significant weight loss prior to presentation.

This case illustrates an atypical and severe presentation of intralobar sequestration complicated by necrotizing pneumonia and sepsis in a previously healthy young adult. While prior reports have described lung abscess and empyema in association with sequestration, progression to necrotizing pneumonia with systemic inflammatory response is less commonly reported, particularly as the initial manifestation leading to diagnosis [9,10]. This highlights the importance of maintaining a broad differential diagnosis in patients with atypical or recurrent pulmonary infections, especially when imaging reveals cystic lesions or aberrant vascular supply.

Definitive management of pulmonary sequestration is surgical resection, particularly in symptomatic patients or when infection is present [8]. However, the timing of intervention remains an important consideration. In this case, surgical resection was deferred to allow for resolution of active infection and inflammation, thereby reducing the risk of intraoperative complications, including hemorrhage from aberrant systemic vessels and postoperative infectious sequelae such as empyema. This approach is supported by the existing literature, which emphasizes stabilization before definitive intervention [11]. Resection has traditionally been performed via thoracotomy, though video-assisted thoracic surgery has produced similar outcomes in select patients [12].

Overall, this case underscores the importance of early recognition of congenital pulmonary anomalies in atypical presentations, the role of imaging in establishing diagnosis, and the need for a multidisciplinary approach to optimize management.

## Conclusions

In this case, intralobar pulmonary sequestration presented as necrotizing pneumonia with sepsis in a previously healthy young adult, an uncommon and severe initial manifestation. Recognition of atypical imaging findings, particularly the presence of systemic arterial supply, was essential in establishing the diagnosis. Initial stabilization with broad-spectrum antibiotics followed by delayed surgical resection resulted in a favorable outcome. The absence of microbiologic confirmation, potentially influenced by prior antibiotic exposure, and the single-patient nature of this report limit the generalizability of these findings. This case highlights the importance of considering congenital pulmonary anomalies in patients with unexplained or severe pulmonary infections and supports a staged management approach when significant inflammation is present at the time of diagnosis.
